# An Unusual Coexistence of Primary Central Nervous System Non-Hodgkin's Lymphoma and Acute Promyelocytic Leukemia

**DOI:** 10.1155/2018/2741939

**Published:** 2018-12-19

**Authors:** Nicola Stefano Fracchiolla, Mariarita Sciumè, Giulia Cernuschi, Agostino Cortelezzi

**Affiliations:** ^1^Nicola Stefano Fracchiolla, Mariarita Sciumè, Agostino Cortelezzi, Hematology Unit, Fondazione IRCCS Ca'Granda Ospedale Maggiore Policlinico, Via Francesco Sforza, 35, 20122 Milan, Italy; ^2^Giulia Cernuschi, Department of Medicine, Fondazione IRCCS Ca'Granda Ospedale Maggiore Policlinico and University of Milan, Via Francesco Sforza, 35, 20122 Milan, Italy

## Abstract

Acute promyelocytic leukemia and primary central nervous system lymphoma are uncommon hematological malignancies. The co-occurrence of acute myeloid leukemia with various lymphoproliferative diseases is an extremely rare condition, especially in the absence of previous chemotherapy or radiotherapy. Herein, we provide a comprehensive characterization of a patient with concomitant diagnosis of extranodal high-grade non-Hodgkin B-cell neoplasm confined to the central nervous system and acute promyelocytic leukemia. We describe the efficacy and feasibility of the consecutive use of all-trans retinoic acid and arsenic trioxide-containing regimen for the treatment of promyelocytic leukemia and high-dose methotrexate plus cytarabine to treat lymphoproliferative involvement of the central nervous system.

## 1. Introduction

Acute promyelocytic leukemia (APL) is a rare variant of acute myeloid leukemia (AML) with an age-adjusted annual incidence of 0.23 per 100,000 persons and a median age at diagnosis of 44 years [1, 2]. Standing the high rate of early mortality due to hemorrhage, it is critical to start treatment with a differentiating agent without delay as soon as the diagnosis is suspected [2].

Primary central nervous system lymphoma (PCNSL) is a rare subtype of extranodal non-Hodgkin lymphoma that involves the brain, leptomeninges, eyes, or spinal cord without evidence of systemic disease. This disease accounts for approximately 3–4% of newly diagnosed central nervous system (CNS) tumours [3–5]. The age-adjusted incidence rate has been reported as 0.47 cases per 100,000 persons per year. Most cases of nonacquired immunodeficiency syndrome-related PCNSL are diagnosed in patients in the fifth decade. The majority of PCNSL are the highly aggressive diffuse large cell subtypes, usually of B-cell phenotypic origin. [3–5].

Here, we describe the case of a patient with acute promyelocytic leukemia who had also a concomitant diagnosis of PCNSL. To our knowledge, there are no published case reports of this very uncommon association.

The followed procedures were in accordance with the ethical standards of the responsible committee on human experimentation (institutional or regional) and with the Helsinki Declaration of 1975, as revised in 1983.

## 2. Case Presentation

A 59-year-old man was admitted to the neurology department in September 2014 with a four-month history of confusion, dysarthria, and progressive deterioration in deambulation capability.

He had no medical history and was unaware of any familial medical problems.

**Figure 1:**
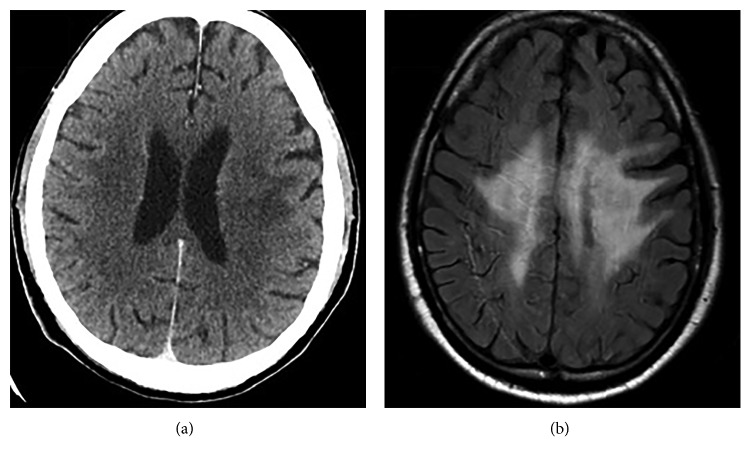
(a) Computed tomographic scan showed a focal lesion in the white matter of the left cerebral hemisphere. (b) FLAIR magnetic resonance imaging sequences showing lesion with surrounding cerebral edema that involved posterior central cerebral convolution, posterior limb of internal capsule, corpus callosum of the left hemisphere, and right central cerebral convolution.

**Figure 2:**
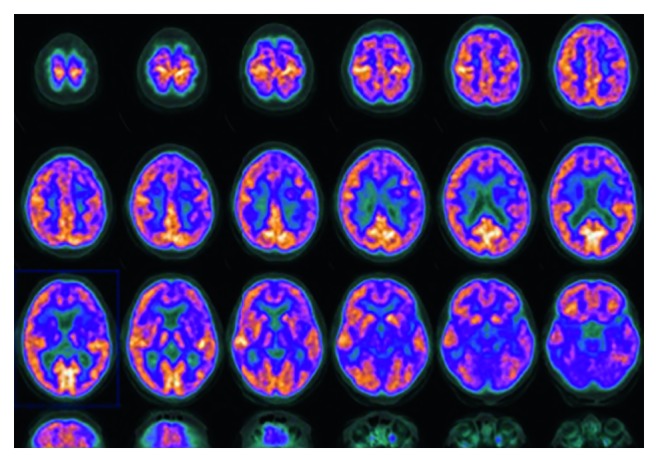
Positron emission tomography scan showing hypermetabolism in the left paraventricular region of the frontal lobe.

On admission, his hemoglobin level was 13.5 g/dl, leucocytes count was 9 × 10^9^/L with normal leukocyte differential count, and platelet count was 120 × 10^9^/L. Coagulation tests showed normal values with a fibrinogen level of 237 mg/dl and D-dimer test 698 *μ*g/ml. Common liver and renal function tests (albumin, bilirubin, alanine transaminase, aspartate aminotransferase, gamma-glutamyl transferase, alkaline phosphatase, and creatinine) were in a normal range. Antibodies directed against human T-cell lymphotropic virus type 1 and human immunodeficiency virus I/II were negative. The cytomegalovirus and Epstein–Barr virus serology showed a pattern compatible with a past infection. Physical examination revealed hyposthenia of the right side of the body. For this reason, a computed tomography (CT) scan was performed, and it highlighted a focal lesion with enhanced border, measuring 15 mm, in the white matter of the left cerebral hemisphere (Figure 1(a)). Magnetic resonance imaging (MRI) of the brain confirmed the presence of a lesion with surrounding cerebral edema that involved posterior central cerebral convolution, posterior limb of internal capsule, corpus callosum of the left hemisphere, and right central cerebral convolution (Figure 1(b)). A positron emission tomogram (PET) scan also showed fludeoxyglucose (FDG) uptake in the left lateral paraventricular region (SUVmax 11.2) (Figure 2). A lumbar puncture was performed, but no additional information was provided.

During the hospital stay, blood examination showed appearance of leukocytosis (white blood cells, WBC 20 × 10^3^/*μ*L) and thrombocytopenia (platelets 24 × 10^3^/*μ*L); coagulation tests demonstrated normal activated partial thromboplastin time ratio, prothrombin time ratio, and fibrinogen level; D-dimer was 2426 *μ*g/ml.

The morphological examination of peripheral blood showed promyelocytes with Auer bodies; flow cytometric analysis demonstrated CD11b + CD11c + CD15 + CD33 + CD38 + CD64 + CD13 + CD2 + CD117 + blast cells, while HLA-DR, CD34, or CD56 were not expressed. Diagnosis of high-risk classic APL was confirmed by cytogenetic [46, XY, *t*(15; 17) (q24; q21) [4]/46, XY, *t*(15; 17) (q24; q21), del(9) (q21q23) [11]/46, XY [2]] and molecular biology tests (PML-RARA/bcr3 fusion transcript). The patient was then transferred to our Hematology Department for the treatment of APL.

In October 2014, in consideration of the APL diagnosis with a suspected CNS involvement, all-trans retinoic acid (ATRA) 45 mg/m^2^ and intravenous arsenic trioxide (ATO) 0.15 mg/kg daily were administered through an off-label use. The therapy was conducted for four weeks plus prednisone (40 mg daily) to prevent ATRA-ATO-mediated differentiation syndrome, obtaining normalization of blood counts. A neurological improvement was also reported. A bone marrow aspirate performed after a month of ATRA-ATO treatment confirmed molecular remission of APL.

Nevertheless, at the time of interruption of steroid therapy, hyposthenia of the right side of the body and dysarthria reappeared and worsened. We repeated a CT scan and an MRI with gadolinium contrast medium that revealed a new hyperdense circular lesion in the white matter of the left frontal lobe.

The PET scan of the brain confirmed hypermetabolism in the left paraventricular region of the frontal lobe that was presumed to be more likely a glial tumor than a lymphoproliferative lesion.

A magnetic resonance spectroscopy (MRS) was performed with the aim of improving the differentiation of locally infiltrative brain tumor from other types of well-circumscribed intracranial lesions by analyzing the chemical composition in a selected area. The MRS revealed an increase of spectroscopic signals of lactate and a reduction in N-acetylaspartate and choline. These data supported the hypothesis of cerebral localization of APL, but the presence of contrast enhancement and the increase of lactate signal might also support the possibility of an immune reconstitution inflammatory syndrome (IRIS). The lumbar puncture showed normal pressure of cerebrospinal fluid, normal values of glucose and proteins, and only 2 white cells/*μ*L; the viral research on cerebrospinal fluid was negative.

In agreement with neurologists, neuroradiologists, and neurosurgeons, we decided to perform a stereotactic biopsy of the cerebral lesion.

The histological sample was diagnostic for primary central nervous system large B-cell lymphoma. Immunohistochemistry identified large cells that were CD20 + BCL-6 ± PAX5 + CD10-MUM1 + CD5-CD3-TdT-MPO- with a Ki-67 index of 80–90%, confirming the presence of B-cell lymphoma. Therefore, on 21 November 2014, we started the first cycle of chemotherapy with high-dose methotrexate and cytarabine (methotrexate 3.5 g/m^2^ on day 1 and cytarabine 2 g/m^2^ every 12 hours on days 2-3) and continued high-dose steroidal therapy (dexamethasone 8 mg b.i.d.).

The chemotherapy was well tolerated, and the hospitalization was complicated by fever of unknown origin treated with empirical antimicrobial therapy. A detailed summary of the main laboratory values is presented in Table 1.

**Figure 3:**
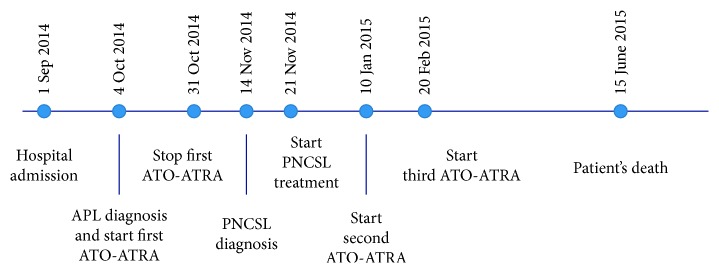
Summary of APL and PCNSL treatment dates.

The patient experienced a gradual improvement of neurological status and was discharged after two months of hospitalization. Subsequently, for family reasons, the patient was transferred to another hospital where he completed the ATRA-ATO program, but no further treatment for PCNSL was performed due to the progressive worsening of clinical conditions. Detailed dates of APL and PCNSL treatments are summarized in Figure 3. The patient died on June 2015 for PCNSL progression in APL complete remission.

## 3. Discussion

The simultaneous occurrence of AML with various lymphoproliferative diseases without prior exposure to chemotherapy or radiotherapy is extremely rare; to our knowledge, we describe for the first time the clinical features and therapeutic management of a patient in which APL and PCNSL developed synchronously.

In this case, the diagnosis of APL was evident because of the appearance of leukocytosis associated with thrombocytopenia and the identification of abnormal promyelocytes and the PML/RARA gene rearrangement in the bone marrow [6]. The PCNSL diagnosis was more difficult because of no classical neuroradiological imaging and absence of peculiar features of cerebrospinal fluid.

Before concluding that neurological impairment was not associated with APL (cerebral localization or IRIS), a stereotactic biopsy brain parenchyma was mandatory [7].

APL is a subtype of AML with specific clinical and biologic features; its genetic hallmark is the balanced reciprocal translocation *t*(15; 17) (q22; q11-12), leading to the resulting PML-RARA hybrid oncoprotein which is responsible for the block of differentiation of leukemic promyelocytes [6].

Pathogenesis of PNCSL is not as clear; different molecular and genetic alterations may contribute to malignant transformation, including aberrant somatic hypermutations of proto-oncogenes, DNA methylation of tumor suppressor genes, gains and losses of genetic material, as well as activation of the NF-*κ*B and JAK/STAT signaling pathway [5]. In our patient, it seems probable that two independent oncogenic processes established in the same period leads to two independent malignancies.

Treatment regimens for concomitant hematological malignancies are not fully established.

Because of the extreme particularity of our clinical case and the documented ability of ATO to cross the blood-brain barrier and penetrate into the cerebrospinal fluid, we decided to treat APL with an off-label ATO-ATRA combination [8–10]. ATO is now licensed in Europe and the United States for the treatment of newly diagnosed low-/intermediate-risk APL in combination to ATRA or for relapsed/refractory patients, and in these settings, it is currently considered to be the therapy of choice due to its high antileukemic activity and low toxicity [11, 12].

We identified PCNSL after APL diagnosis, so firstly, treatment with ATO-ATRA was started, followed by a classical first-line therapy for PCNSL based on combination chemotherapy with high-dose methotrexate and cytarabine [5]. Consecutive administration of these therapeutic regimens was well tolerated with APL molecular remission already after the induction phase. For safety reasons, treatments of APL or PCNSL were not administered together.

Interestingly, the specific therapies used for the two oncohematological diseases might influence the course of each other. Steroid therapy used to prevent ATRA-ATO-mediated differentiation syndrome led to a neurological improvement; corticosteroids are a well-known component of PCNSL treatment protocols because of their direct apoptotic effect on lymphoma cells [5]. On the contrary, combination of methotrexate and cytarabine used for PCNSL could have a potential beneficial effect on APL. Cytarabine is still part of the standard chemotherapy program of high-risk APL, and methotrexate has a role in APL maintenance treatment [6].

We suggest that if clinicians had to treat two synchronous oncohematological diseases, they should firstly privilege treatment of the pathology associated with major immediate life risks.

In conclusion, we described the rare concomitance of APL and PCNSL in a patient who was not previously exposed to genotoxic agents. Furthermore, we showed the feasibility of consecutive use of ATO-ATRA regimen for the cure of APL and high-dose methotrexate plus cytarabine to treat PCNSL.

## Figures and Tables

**Table 1:** Laboratory values in the main time-points from hospital admission to PNCSL diagnosis.

	Admission 1 Sep 2014	7 Sep 2014	15 Sep 2014	1 Oct 2014	APL diagnosis and start of ATO-ATRA + prednisone 4 Oct 2014	After 7 days of ATO-ATRA + prednisone 11 Oct 2014	After 15 days of ATO-ATRA + prednisone 20 Oct 2014	At the end of ATO-ATRA induction + prednisone 31 Oct 2014	At PNCSL diagnosis 14 Nov 2014
Hb (g/dl)	13.5	13	13	13.3	13.4	10	8.8	10.1	12.6
WBC count (×10^9^/L)	9	9.5	15	10	20	20.9	5.7	3.2	8.8
ANC (×10^9^/L)	2.4	2.5	4.5	2.5	2.5	17.5	3.4	1.4	5.8
Blast cells (%)	0	10	60	64	70	11	12	0	0
Platelets (×10^9^/L)	120	90	24	15	47	47	24	361	429
GOT (U/L)	20	19	19	17	15	64	49	35	16
GPT (U/L)	30	32	29	29	30	125	129	63	15
Creatinine (mg/dl)	0.9	0.9	0.9	0.9	0.9	0.7	0.6	0.5	0.6
Fibrinogen (mg/dl)	237	240	250	237	275	207	214	289	536
D-dimer (*μ*g/ml)	698	690	2430	2400	9903	707	862	641	492
LDH (U/L)	240	230	300	309	304	448	227	186	195
PT (ratio)	1.06	1.07	1.05	1.07	1.13	0.95	0.92	1.01	1.03
aPTT (ratio)	0.87	0.89	0.85	0.88	0.91	0.78	0.78	0.9	1.07

Hb: hemoglobin (normal values 13.5–17.5 g/dl); WBC: white blood cell (normal values 4.8–10.8 × 10^9^/L); ANC: absolute neutrophil count (normal values 1.5–6.5 × 10^9^/L); GOT: aspartate transaminase (normal values 5–38 U/L); GPT: alanine aminotransferase (normal values 5–41 U/L); creatinine normal values 0.5–1.2 mg/dl; fibrinogen normal values 165–350 mg/dl; D-dimer normal values 0–230 *μ*g/ml; LDH: lactate dehydrogenase (normal values 135–225 U/L); PT: prothrombin time (normal values 0.88–1.21 sec); aPTT: partial thromboplastin time (normal values 0.85–1.18 sec).
